# 

**Published:** 1887-07-02

**Authors:** 


PRICE ONE PENNY.

No. 40, Vol. II.

July 2nd, 1887.

Registered at the General Post Office as a Newspaper.]

Per Annum, 6s. 6d., post free.
Monthly Parts, 6d.; post free, 7½d.
Ditto per Ann., 7s. 6d., post free.

